# Automated Segmentation of Infarct Lesions in T1-Weighted MRI Scans Using Variational Mode Decomposition and Deep Learning

**DOI:** 10.3390/s21061952

**Published:** 2021-03-10

**Authors:** May Phu Paing, Supan Tungjitkusolmun, Toan Huy Bui, Sarinporn Visitsattapongse, Chuchart Pintavirooj

**Affiliations:** 1 School of Engineering, King Mongkut’s Institute of Technology Ladkrabang, Bangkok 10520, Thailand; 59601030@kmitl.ac.th (M.P.P.); supan.tu@kmitl.ac.th (S.T.); 2 Course of Science and Technology, Graduate School of Science and Technology, Tokai University, Tokyo 108-8619, Japan; 9ltad006@mail.u-tokai.ac.jp

**Keywords:** brain infarction, stroke, U-Net, variational mode decomposition

## Abstract

Automated segmentation methods are critical for early detection, prompt actions, and immediate treatments in reducing disability and death risks of brain infarction. This paper aims to develop a fully automated method to segment the infarct lesions from T1-weighted brain scans. As a key novelty, the proposed method combines variational mode decomposition and deep learning-based segmentation to take advantages of both methods and provide better results. There are three main technical contributions in this paper. First, variational mode decomposition is applied as a pre-processing to discriminate the infarct lesions from unwanted non-infarct tissues. Second, overlapped patches strategy is proposed to reduce the workload of the deep-learning-based segmentation task. Finally, a three-dimensional U-Net model is developed to perform patch-wise segmentation of infarct lesions. A total of 239 brain scans from a public dataset is utilized to develop and evaluate the proposed method. Empirical results reveal that the proposed automated segmentation can provide promising performances with an average dice similarity coefficient (DSC) of 0.6684, intersection over union (IoU) of 0.5022, and average symmetric surface distance (ASSD) of 0.3932, respectively.

## 1. Introduction

Brain infarction, generally known as stroke, is a global health issue and public health priority. It is a significant cause of disability and the second leading cause of death worldwide [1]. Based on the up-to-date statistical data from the World Stroke Organization (WSO), over 13.7 million new cases and 5.5 million deaths of stroke are occurring annually [2]. Moreover, up to two-thirds of stroke survivors usually suffer residual disabilities and no longer participate in their daily activities [3]. Examples of disabilities may include transient or lasting paralysis on one or both sides of the body, difficulties in speaking or eating, and muscular coordination loss. Such devastating and life-altering results after brain infarction can seriously impact a critical economic and humanistic burden as well [1]. Approximately an annual $51.2 billion economic loss results from stroke-reducing approaches, for example, medical costs and costs for rehabilitation in poststroke patients such as physical functioning and caregiver involvement [4].

In medical terminology, “infarction” is also known as “necrosis.” It is damage or death of tissues due to the failure of blood and oxygen supply to the affected area. Brain infarction or stroke is a type of infarction that mainly affects the brain. Specifically, it is a cerebrovascular disease resulting from the formation of necrotic or damaged tissues inside the brain. It commonly occurs when an artery in the brain gets blocked by clots (ischemic stroke) or bursts (hemorrhagic stroke) [5]. Fortunately, brain infarction is curable if it is promptly detected after it occurred. Thrombolysis, which is a treatment of dissolving the blood clots and restoring the blood flow to the brain, is generally used to cure ischemic infarction. However, prompt time is critical, and it should be performed within 4.5 h. Similarly, hemorrhagic also needs emergency surgery to stop the bleeding in the brain. For those problems, early detection, prompt actions, and immediate treatments are essential in reducing disability and death risks by stroke.

Neuroimaging technologies such as magnetic resonance imaging (MRI) and computed tomography (CT) scans are standard modalities to diagnose the brain infarction. Compared to CT, MRI is more commonly used in clinical practice because it can expose a more precise representation of brain lesions such as soft-tissues, nerves, ligaments, and muscles [6]. However, to date, manual lesion tracing by expert neurologists remains the gold standard for infarct lesion segmentation in practice. They read the individual brain scans slice by slice and delineate the infarcted brain lesions. Indeed, this manual delineation process is tremendously tricky due to the similar appearance but ambiguous boundaries between normal and infarct lesions. Besides, it is effort-intensive and time-consuming to distinguish the complicated structures such as shape, size, and texture of brain lesions. A study conducted by [7] stated that the manual segmentation of abnormal lesions from the neuroimaging scans takes between 4.8 and 9.6 h. As a negative consequence, manual diagnosis ultimately becomes a key impediment for urgent clinical decisions and stroke treatments. Therefore, a quick, accurate, and automatic diagnosis scheme is essential to provide prompt and reliable therapy for stroke patients.

## 2. Related Works

In neuroimaging analysis, several studies for automated delineation of brain infarct lesions from MRI scans have emerged. Generally, previous studies that were conducted at the beginning of the last decade were based on the standard machine learning algorithms such as K Nearest Neighbors (KNN) [8], Naive Bayes (NB) [8,9], Support Vector Machine (SVM) [6,10,11], Random Forest (RF) [12,13,14], and so on. These conventional techniques were simple and easy to use; however, their major weakness is that the performance was strongly dependent on the quality of handcraft features. Extracting meaningful features from the images is crucial to make the machine learning models learnable and robust [15]. In practice, handling of such features is very time-consuming and challenging because machine learning engineers are not medical domain experts.

With the state-of-the-art advancements and substantial results of convolutional networks, there is no doubt that deep learning algorithms hit a milestone in medical image analysis. Unlike the standard machine learning techniques, deep learning-based methods are not necessary to extract handcrafted features. They can learn high-level features directly from the input images and can provide more reliable results. Due to these advantages, the deep learning-based methods became trendier in automated diagnosis of brain infarction. For example, the use of benchmark deep learning models such as AlexNet, VGG, Inception, ResNet, are found as transfer learning algorithms for infarct lesion detection and classification [16,17,18]. Although they are applicable for detection and classification tasks, they need to follow a general encoder-decoder architecture to perform a semantic segmentation. More specifically, such standard deep networks can be applied as encoders to extract the discriminative features from the inputs and to perform pixel-wise classification. However, due to the use of constitute convolutions in encoder networks, the resolution of the inputs becomes lower and cannot produce the segmentation results having the same dimension as the input. For this problem, a decoder network is necessary to upsample and enhance the resolution of the convoluted images. Based on a recent and intensive review of deep learning method for neuroimaging, it is found that fully connected network (FCN) and U-Net were widely used for semantic segmentation of infarct lesions. Both methods follow encoder-decoder structure, but FCN based semantic segmentations have only one upsampling layer in the decoder part and mainly use bilinear interpolation for upsampling. Unlike FCN, U-Net’s architecture is designed using multiple upsampling layers along with skip-connections and concatenations. Moreover, it uses learnable weight filters instead of fixed interpolation for upsampling. This architecture makes U-Net more robust and provides better segmentation results compared to conventional FCN based segmentations. For these strengths, we choose U-Net over other deep learning models in this study.

Brain infarct lesions segmentation based on U-Net architecture was the most frequently used method in recent studies [19,20,21,22,23,24,25]. It is a baseline and famous state-of-the-art deep learning architecture in biomedical image segmentation. Depending upon the modification of the U-Net architecture, the names of the segmentation Nets were changed study by study. Among [19,20,21,22,23,24,25], Cross-Level fusion and Context Inference Network X-Net [21], (CLCI-Net) [22], Deep Residual Attention Convolutional Neural Network (DRANet) [23] used two-dimensional (2D) based U-Net architectures. They segmented the infarct lesions from input 2D slices of the MRI based on a single orientation. As a weakness, the performance of such 2D-based methods is limited because they cannot access the spatial information of the lesions from the other two planes. Moreover, those methods also required extensive postprocessing mechanisms to combine slice-by-slice predictions into final volumetric segmentation outputs.

Unlike the 2D-based U-Nets, multi-path 2.5D CNN [24] considered the volumetric information of the brain lesions by performing three different normalizations for each of the three axial, sagittal, and coronal planes. Nine different 2D paths resulted from the normalizations were then fed into the nine end-to-end U-Nets, and path-wise segmentations were performed. However, like the aforementioned 2D-based U-Nets, the 2.5D net also had to perform an extensive postprocessing task. It used 3D CNN to concatenate 2D lesion masks for postprocessing.

Apart from the previous 2D and 2.5D U-Nets, fully 3D architectures were also found in 3DCRF [19], D-UNet [20], and 3D-Res-UNet [25]. Since these U-Nets worked on the volumetric inputs in 3D space, they can fully utilize the contextual and spatial information of the infarct lesions to provide more robust predictions. However, as a tradeoff, these fully 3D models significantly spend more computational resources in training.

The primary purpose of this paper is to present an alternative and automatic scheme to segment infarct lesions from brain MRI scans. Like the previous 3D-based methods, our proposed approach is also based on volumetric segmentation using U-Net. However, as a difference, the automated infarct lesion segmentation proposed in this paper applies variational mode decomposition (VMD) followed by a three-dimensional U-Net-based segmentation. VMD is a popular preprocessing method, and its efficacy in brain MRI analysis had been proposed in [6,11,26]. However, all those studies utilized conventional machine learning algorithms, specifically support vector machine (SVM), combined with VMD to classify normal and abnormal brain lesions. To our knowledge, the use of VMD together with a deep learning model has not been conducted in previous brain abnormalities detections. Indeed, deep learning-based methods obviously outperform traditional machine learning algorithms, and it has been proven by several technical studies. For this reason, we decided to apply VMD and U-Net model in order to take advantage of both methods.

Our proposed method brings three significant technical contributions as follows:

(i) For the first contribution, we proposed variational mode decomposition (VMD) as a preprocessing task. It helps remove non-infarct tissues from the input MRI scans and lessens the amount of unwanted information from the input volumes.
(ii) For the second contribution, we presented overlapped patches strategy, which divides the input MRI volumes into smaller patches. The divided patches were fed into the U-Net model to perform patch-wise segmentation. The proposed overlapped patch strategy also performed patch pruning to reduce the workload of the segmentation model. Moreover, it records the reference numbers of patches aiming at seamless and intensive postprocessing.
(iii) For the last contribution, we developed a three-dimensional U-Net model for the segmentation of infarct lesions from volumetric patches. Then, a postprocessing was followed in order to produce the final segmentation results.

The rest of the paper is organized as follows: Section 3 will discuss the details about the materials and methods applied in this study. Section 4 will explain the experimental results and discussion, and finally, Section 5 will summarize and conclude the paper.

## 3. Materials and Methods

### 3.1. Overview of the Proposed Method

**Figure 1:**
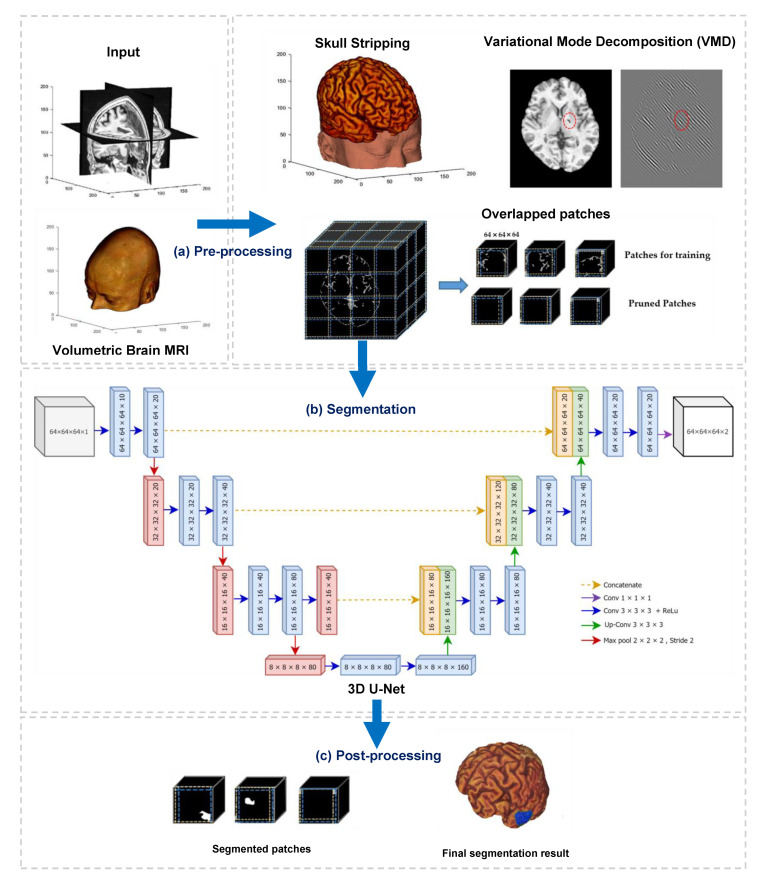
Overview of the proposed method, which contains three main processes: (**a**) preprocessing, (**b**) segmentation, and (**c**) postprocessing.

This paper developed a deep learning-based algorithm for the segmentation of infarct lesions from chronic-stroke MRI scans. Figure 1 demonstrates an overview of the proposed method, and it consists of three fundamental processes, namely preprocessing, segmentation, and postprocessing.

The primary objective of preprocessing in this study is to reduce the computational workload by suppressing and removing unwilling parts from the input images, for instance, background, skull, and other non-infarct tissues. We conducted three main operations in the preprocessing step. They are (i) stripping of the skull using a pretrained model, (ii) removing non-infarct lesions using variational mode decomposition (VMD), and (iii) dividing the output volumes of VMD into small patches using overlapped patches strategy. The outputs of preprocessing step are three-dimensional patches of brain scans and associated lesion masks. In the second process, segmentation, the divided patches are subsequently fed into a three-dimensional U-Net model to perform patch-wise semantic segmentation. Finally, a postprocessing step is followed to combine the segmented patches and generate the segmented infarct lesions.

### 3.2. Data Source

The brain MRI scans applied in this study are obtained from a freely accessible and standard dataset called Anatomical Tracings of Lesions After Stroke (ATLAS) [3]. The raw images in the dataset were collected from chronic-stroke patients in 11 cohorts worldwide. There was a total of 304 T1-weighted MRIs in the original version of the dataset. Along with the dataset, manually delineated lesion masks and metadata can also be downloaded for the ground truths. The reliability of the lesion masks in ATLAS dataset were thoughtfully reviewed and confirmed by an expert radiologist. The individual subject of MRI contains at least one lesion, and 58% of subjects in the dataset have a single lesion. The rest, 42.1%, are multiple lesions, and separate lesion masks were used to identify them.

Besides the original raw MRIs, ATLAS also provides a standardized version of the dataset. This standard version was created to reduce the technical difficulties due to the image quality produced by different scanners. Some MRI subjects, especially collected using 1.5 T scanner, were removed from the original raw dataset (containing 304 T1-weighted MRIs), and the rest were defaced, normalized to standard MNI-152 space. As a result, there were a total of 239 scans in the standard ATLAS dataset. In this study, we will apply the standard ATLAS dataset to conduct the experiments. Each input MRI in the standard dataset has a dimension of 197 × 233 × 189 mm^3^ with a canonical voxel size of 1 mm^3^.

### 3.3. Variational Mode Decomposition (VMD)

Variational mode decomposition (VMD) is one of the most popular decomposition methods in biomedical image analysis. It decomposes an image into a specific number of spectral bands, having different directional and oscillatory characteristics. As a result of decomposition, VMD produces a discrete number of modes in which each mode has limited bandwidth around its center frequency. For instance, suppose a two-dimensional input image f(x) is decomposed into k number of modes using VMD. The spatial bandwidths in each mode k are needed to be compact around a center pulsation ωk [27]. To calculate the bandwidths in each mode uk, analytical signals of each mode should be computed first using the following equations.

(1) $$min_{u_{k},\;\omega_{k}}\left\{\sum_{k}\propto_{k}||\nabla \left[u_{AS,\;k}\;\left(x\right)\;e^{-j\left(\omega_{k},x\right)}\right]|{|}_{2}^{2}\right\},$$

(2) $$s.t.\;\forall \;x\;:\;\underset{k}{\sum }u_{k\;}\left(x\right)\;=\;f\left(x\right)$$

where ∝ is the bandwidth constraint and $u_{AS,\;k}\left(x\right)$ represents the analytic signal of the *k*^th^ mode.

However, the objective function in Equation (1) has a reconstruction constraint because it was calculated by setting one half-plane of the frequency domain to zeros. Therefore, quadratic penalty and Lagrangian multiplier are conducted to render this constraint. Finally, the optimal mode uk of the image can be obtained using the following equations [26,27].

(3) $$min_{u_{k},\;\omega_{k}}max_{\lambda }ℒ\;\left(\left\{u_{k}\right\},\;\left\{\omega_{k}\right\},\lambda \right),$$

where ℒ in Equation (3) is the augmented Lagrangian, and the saddle point of ℒ is the solution to the original constraint minimization problem. *λ* is the Lagrangian multiplier term, and the following Equation (4) can be derived to change into the quadratic penalty term,

(4) $$\underset{k}{\sum }\propto_{k\;}||\nabla \left[u_{AS,\;k}\;\left(x\right)\;e^{-j\left(\omega_{k},x\right)}\right]|{|}_{2}^{2}\;+||\;f\left(x\right)\;-\;\underset{k}{\sum }u_{k\;}\left(x\right)\;+\;\frac{\lambda \;\left(x\right)}{2}|{|}_{2}^{2}-||\frac{\lambda \;{\left(x\right)}^{2}}{4}|{|}_{2}^{2},$$

**Figure 2:**
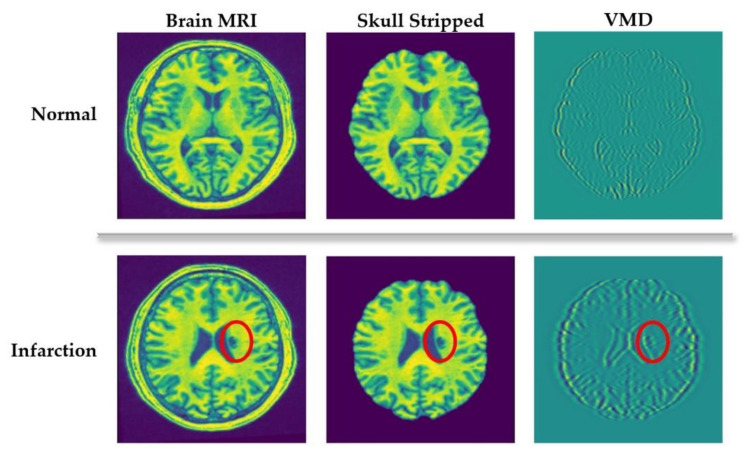
Variational mode decomposition (VMD) of healthy and abnormal (infarcted) brain scans.

The main idea of applying VMD in this study is to extract silent image features from the spectral characteristics of the decomposed images. For a clear understanding, a visual representation can be seen in Figure 2. It compares the VMD of normal and infarcted (denoted by the red circle) brain MRI scans. As we can see in Figure 2, VMD transforms the input images into a number of spectral bands exposing different directions and oscillatory characteristics. Such spectral characteristics are the key indicators of distinctive anatomical features, which are very useful for further diagnostical analysis. In the second row of Figure 2, it can be evidently seen that the spectral bands around the abnormal (infarct) lesion exposes higher oscillations compared to other areas. Based on this fact, we create candidate infarct lesion masks by suppressing the mode oscillation values. Applying these masks, the objects showing low potential to be infarct lesions can be removed.

### 3.4. Overlapped Patches Strategy

Deep learning for medical images is notably arduous when the input images are volumetric data obtained from a stack of multiple sequential 2D images. The overlapped patches strategy proposed in this research intends to reduce the training efforts and time by dividing the input volume into smaller patches. As described in Section 3.2, the dimension of each input MRI is 197 × 233 × 189 mm^3^ with a canonical voxel size of 1 mm^3^. Feeding the whole volume into the deep learning-based segmentation model is very bulky and computationally intensive. For this reason, our proposed overlapped patches strategy tries to create smaller, same-dimensioned patches before segmentation. Note that the MRI volumes in this stage were resulted from skull stripping and VMD decomposition, as illustrated in Figure 1. Since the skull stripping and VMD do not affect the input MRIs’ dimension, the resulted volumes after passing those operations remain the same as the original input volume (197 × 233 × 189 mm^3^).

These skull-stripped and VMD-masked volumes are divided into small, overlapped patches using the overlapped patches strategy. Each patch has 64 × 64 × 64 in dimension, and ten voxels overlapped to its adjacent patches. A zero-padding of the original volume (197 × 233 × 189) is conducted to exactly divide the input volume into 64 × 64 × 64 patches. Thus, after padding, the volume size became 256 × 256 × 192. Therefore, every input volume MRI generates the same number of patches (48 patches in total). The corresponding annotation masks are also divided into patches. Moreover, the reference numbers of patches for each input subject are also recorded, aiming for seamless, intensive stitching in the postprocessing stage. We used the subject_ID and a serial number of the patch to record the reference numbers—for example, “c0003_patch_1” means the very first patch of the input c0003.

**Figure 3:**
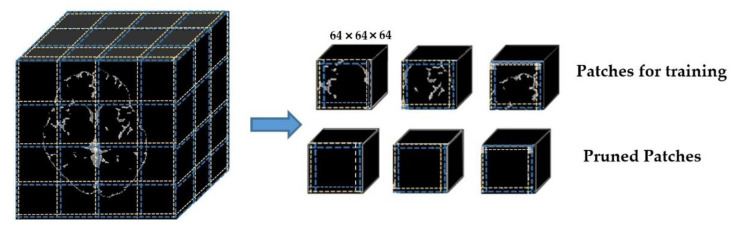
Dividing into overlapped patches.

Although the primary purpose of separating overlapped patches is to reduce the volume size and computation effort, the padding makes the volume size bigger. Thus, our proposed overlapped patches strategy alleviates this problem by pruning unnecessary patches. Figure 3 demonstrates how the proposed overlapped patches strategy works. If the summation of all voxels in a patch is equal to zero, then that patch does not need to consider for segmentation. Moreover, patch pruning does not hinder the postprocessing thanks to using the same number of patches for every subject and recording the reference number of patches.

### 3.5. Three-Dimensional U-Net (3D U-Net)

U-Net [28] is one of the state-of-the-art deep learning models for semantic segmentation of images, and it has been successfully applied in biomedical image segmentation. As the name implies, the architecture of U-Net exposes a U-shaped structure, which is comprised of two main parts: contracting path (encoder) and expansive path (decoder). The first path, the encoder, extracts the discriminative features from the input images. Specifically, it follows the typical architecture of a convolutional neural network and contains repeated series of two 3 × 3 convolutions, each followed by a rectified linear unit (ReLU) and a 2 × 2 max pooling. Since the goal of the contracting path is feature extraction, the number of feature channels in each downsampling step becomes double while the spatial dimensions are reduced. The bottommost layer of the U-Net is treated as a bridge between contraction and expansive paths, and it contains two 3 × 3 convolution layers followed by ReLU and one 2 × 2 up convolution layer.

U-Net applies a series of convolutional filters in the contraction path; thus, the spatial dimensions of the inputs at the bottommost layer become smaller than that of the original images [20]. Since the ultimate goal of U-Net is semantic segmentation, which is a classification of pixels to determine whether a specific pixel in the input is part of a lesion, the output should be the same dimension as the input. For this reason, the expensive (decoder) path of U-Net semantically projects the discriminative features (lower spatial dimensions) generated by the encoder onto the pixel space (higher spatial dimensions) to maintain symmetric dimensions between input and output images. Similar to the encoder, every step in the decoder path consists of a 2 × 2 upsampling (upconvolution), followed by two 3 × 3 convolutions with ReLU. At the final layer, U-Net ends up with a 1 × 1 convolution that converts the size of the feature map of the first last layer to the desired number of output classes.

**Figure 4:**
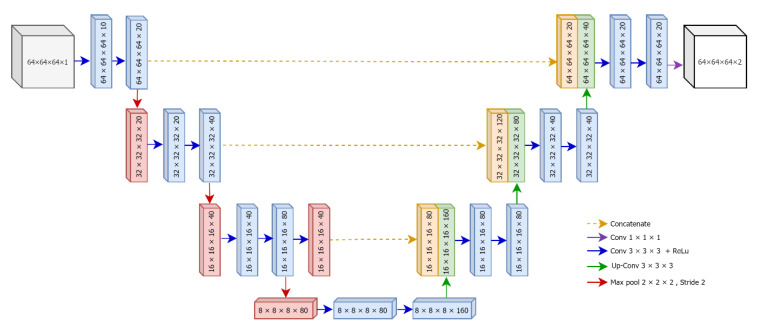
The architecture of proposed 3D U-Net.

The detailed architecture of our proposed 3D U-Net is illustrated in Figure 4. The original version of U-Net was designed for the segmentation of two-dimensional color images. However, in this research, we tend to segment infarct lesions from volumetric patches of brain MRIs. Therefore, we develop a three-dimensional (3D) version of U-Net based using divided patches using overlapped patches strategy.

## 4. Results and Discussion

### 4.1. Configurations of the Proposed Method

Working with deep learning models can guarantee better performance, but they considerably demand a high number of hyperparameters. A correct configuration and the best choice of hyperparameters for the model are the most critical issues to get the accurate outputs. This section will discuss the details of the experimental setups and the results of our proposed method.

#### 4.1.1. Data Preparation

As stated in the materials and methods (Section 3), the experiments of our proposed method are done using 239 MRI exams of the standardized ATLAS dataset. We divided those input data into three partitions: 60% for training (143 scans), 20% for validation (48 scans), and 20% for testing (48 scans). Since some MRI scans contain more than one infarct lesion, we summarized data preparation details in Table 1.

#### 4.1.2. Preprocessing

Once preparing the data, we perform the preprocessing of the input images. Since an input MRI exam is taken from an individual patient and contains multiple two-dimensional (2D) sequential slices, we stacked these 2D slices in sequential order and constructed them as a volumetric image. However, we did not normalize the input volumetric images because we used the standardized ATLAS dataset version. All the exams are already undergone a standardization process and formatted into 197 × 233 × 189 mm^3^ dimensions with a canonical voxel size of 1 mm^3^. For this reason, we skipped the standardization process and continued the following preprocessing steps.

- Skull Stripping

Skull stripping is one of the most initial and crucial tasks in every type of neurological MRI analysis. On a head scan, the brain region occupies approximately one-third of the entire scan while the rest, two-third, is occupied by extra-meningeal tissues. Skull stripping detects the boundaries of the skull to determine the brain area. Subsequently, it removes nonbrain tissues outside of boundaries and extracts the brain region only. In this study, we focus on detecting the infarct brain lesions located within the brain area. Thus, this step is necessary not only to reduce search area and computational effort but also to improve the detection accuracy. Several approaches had been proposed to perform this operation. Among them, we applied a deep learning-based method called DeepBrain [29] for skull stripping. The main reasons for using DeepBrain includes (i) it is developed using T1 weighted MRIs, and we are also working on T1 weighted MRIs, (ii) it is easy to use and fast (only ~20 s in CPU version and ~2 s in GPU version), (iii) it is working well on 3D volumetric data without requiring any extra effort and (iv) it had proven high accuracy (>0.97 dice metric) using popular standard datasets.

- Variational mode decomposition (VMD)

After skull-stripping from the input brain MRIs, the next preprocessing step is variational mode decomposition (VMD). As described in Section 3.3, applying VMD as a preprocessing is one of the major contributions of this study. Our main objective of this contribution is to suppress the non-infarct lesions inside the brain. It can significantly reduce the computation effort and time because the deep learning model does not need to segment such non-infarct lesions. On the other side, VMD also provides a great deal of help in reducing overfitting and data imbalance problems. VMD works on a number of hyperparameters, and we selected the appropriate values of hyperparameters as described in Table 2. Among (K = 5) modes of decomposed images, experiments showed that mode number 3 is the most suitable for decomposition because it can represent most information about infarct lesions. Thus, we applied mode three decomposed images to create masks and applied them to cover out undesired non-infarct lesions.

- Overlapped Patches Strategy

Once masking out the non-infarct candidates, we then divided the resulted volumes into smaller patches. These patches have the same dimensions (64 × 64 × 64), and each patch possesses ten overlapped pixels from its adjacent patches. Moreover, each patch’s reference position number is also extracted simultaneously, aiming for seamless and intensive stitching in the postprocessing step. These divided patches are then fed into the 3D U-Net algorithm in order to perform patch-wise segmentation.

#### 4.1.3. Segmentation Using 3D U-Net

We preprocessed for all MRIs and associated ground-truth scans in the dataset hence each patch has its own associated mask patch. The outputs from the preprocessing step are 3D overlapped patches and corresponding masks, and they were used as the inputs for 3D U-Net based segmentation. As described in the graphical representation of the proposed U-Net (Figure 4), the inputs are 64 × 64 × 64 × 1 patches. Here, the color channel is assigned as one because MRIs are the grayscale images. The output of U-Net is 64 × 64 × 64 × 2 for two classes, that is one for background and another for the infarct lesion.

The proposed 3D U-Net was trained using 3D patches that were obtained from MRIs in training partition. The patches for validation were apart from the training patches and used to evaluate the model performance. Dice loss function is calculated to assess the training performance and Adam optimizer is applied to optimize the loss. Moreover, we applied batch normalization after each layer of convolution to improve the stability of the training and drop-out after each level of U-Net to reduce overfitting. Several times of training using different values of hyperparameters are conducted to get the lowest loss value on the validation samples. Based on the experimental trials and results, we achieved our best segmentation model using the following hyperparameters stated in Table 3.

**Figure 5:**
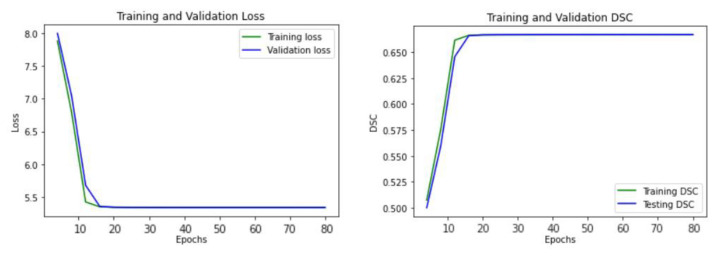
The learning curve of the proposed U-Net model.

Figure 5 illustrates the learning curve of our proposed U-Net model, showing the training and validation loss. The model reached the best stage at epoch 20 with a mean DSC of 0.6738 for training, and 0.6718 for validation, respectively.

#### 4.1.4. Postprocessing

The is the final step of our proposed infarct lesion segmentation. The segmented image patches using 3D U-Net were stitched together again, using the reference patch position numbers and 3D connected component labeling. In preprocessing, we pruned out some patches having all black voxels, and they were not fed into the U-Net for segmentation. Therefore, we substitute zero voxels again for those patches to get the same dimension as the original image. Then, we performed 3D connected component labeling using voxel connectivity 26 to finetuned on full-size images and generate the final segmentation of infarct lesions. In order to get more comprehensive understanding, Algorithm 1 describes the pseudo code to summarize the workflow of our proposed automated infarct lesion segmentation.

### 4.2. Results

To evaluate the performance of the proposed segmentation, we calculated the following assessment measurements. Note that all of these measurements were calculated after the postprocessing stage; that is, they were not calculated for patch-wise segmentation but for the final volumetric lesion segmentation.

- Jaccard similarity coefficient (IoU)

This index is also known as intersection over union (IoU) and measures the overlap between the segmented lesions and the ground truth images. IoU value is ranging from 0 (no overlapped) to 1 (perfect segmentation), and it can be calculated using the following equation:

(5) $$\mathrm{IoU}\;\left(X,Y\right)=\;\frac{\left|X\;\cap^{\;}Y\right|}{\left|X\;\cup^{\;}Y\right|},$$

where *X* is the segmented lesion and *Y* is the ground truth lesion mask.

- Dice similarity coefficient (DSC)

Similar to IoU, DSC also measures the overlapped between the segmented lesion and the ground truth lesion mask. DSC can be calculated by:

(6) $$\mathrm{DSC}\;\left(X,Y\right)=\;\frac{2\left|X\;\cap^{\;}Y\right|}{\left|X\right|+\left|Y\right|},$$

- Average symmetric surface distance (ASSD)

Unlike IoU and DSC, this index is a distance measurement. It calculates the average of all the distances from voxels on the boundary of the segmented lesion to those of the ground truth and vice versa [30]. The smaller number of ASSD indicates the better segmentation performance.
**Algorithm 1.** Pseudo Code for Proposed Infarct Lesion SegmentationInput:Brain MRI exams as S = {s_1_, s_2_, …., s_n_}, and associated ground truth masks as M = {m_1_, m_2_, …., m_n_}, where n is the total number of exams in the given dataset.Step 1:Prepare the data for training, validation, and testing. Assign 60% of the given dataset for training, 20% for validation, and 20% for testing. n_train = n ∗ (60/100) n_val = n ∗ (20/100) n_test = n ∗ (20/100) Divide S and M into training split. S_train_ = {s_1_, s_2_, …., s_n_train_} M_train_ = {m_1_,m_2_,…,m_n_train_} Divide S and M into validation split. S_val_ = {s_n_train +1_, s_n_train +2_, …., s_n_train + n_val_} M_val_ = {m_n_train +1_, m_n_train +2_, …., m_n_train + n_val_} Divide S and M into testing split. S_test_ = {s_n_train+n_val +1_, s_n_train+n_val+2_, …., s_n_train+ n_val+ n_test_} M_test_ = {m_n_train+n_val +1_, m_n_train+n_val+2_, …., m_n_train+ n_val+ n_test_}Step 2:Perform preprocessing of the input MRI exams.       Perform skull-stripping using DeepBrain.                   DeepBrain (S_train_, S_val_, S_test_)                          return S_train_stripped_, S_val_stripped_, S_test_stripped_       Perform variational mode decomposition of skull-stripped exams.                   VMD (S_train_stripped_, S_val_stripped_, S_test_stripped)_                          return S_train_vmd_, S_val_vmd_, S_test_vmd_       Divide the decomposed exams and associated ground truths into       overlapped patches.                   Overlapped Patches (S_train_vmd_, S_val_vmd_, S_test_vmd_, M_train_, M_val_, M_test_)                          return S_train_patches_, S_val_patches_, S_test_patches_                                 M_train_patches_, M_val_patches,_ M_test_patches_Step 3:Develop 3D U-Net model based on the desired architecture.Step 4:Train the U-Net model using S_train_patches_, S_val_patches,_ M_train_patches_, M_val_patches_.Step 5: Test the trained U-Net using S_test_ and perform postprocessing.Step 6:Evaluate the performance of U-Net using M_test_.Output:Segmented infarct lesions of tested MRIs and assessment measurements.

**Figure 6:**
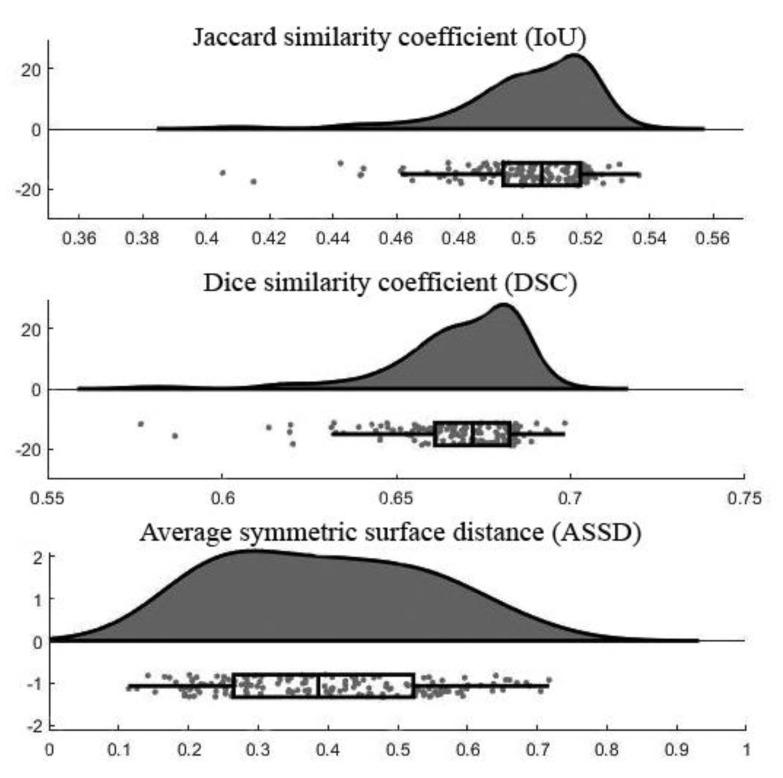
Raincloud plots of assessment measurements.

Figure 6 demonstrates the raincloud plots showing the distribution of three assessment measurements on testing data. By analyzing the raincloud visual representation, it is evident that the plots for IoU and DSC are relatively short, meaning overall segmentation results have a high level of agreement with each other. For the ASSD, the plot is comparatively taller than the others because some outputs had low ASSD and some were high. The mean and standard deviation values of each assessment measurement were summarized in Table 4. Note that the lower number of ASSD value indicates the higher similarity between the segmentation result and ground truth.

### 4.3. Discussion

**Figure 7:**
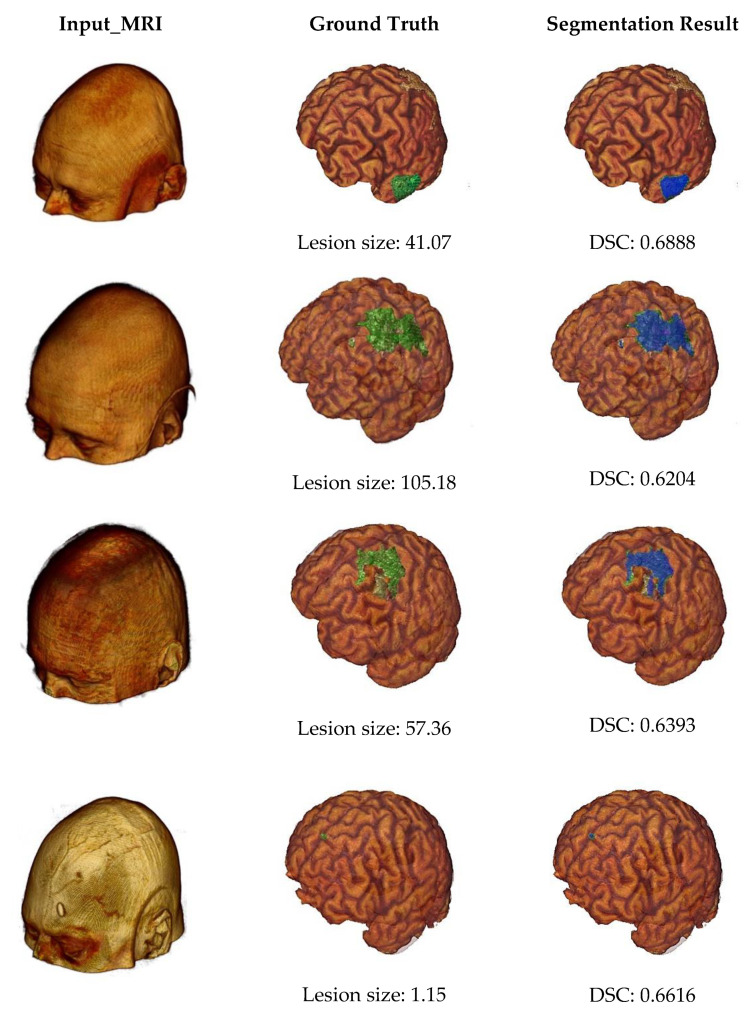
Volume rendering of some example outputs.

Some example outputs of our proposed segmentation method are illustrated in Figure 7 using volume rendering. The first column of Figure 7 represents input MRI scans from four different patients containing different sizes of infarct lesions. Then, in the second column, we can see the skull stripped volumes of input images and associated lesion masks (highlighted by the green color). The lesion size of each subject was also described by measuring the number of voxels in the major axis length. And finally, the last column shows the segmentation results (highlighted by the blue color) and associated DSC values. From this figure, we can note that our proposed automated infarct lesion segmentation performs well for any size of lesions.

Moreover, we evaluated the performance of our proposed method by performing a comparative analysis with state-of-art methods described in related work (Section 2). Comparing different methods that were trained using different datasets and measured using different assessments is very troublesome. For that reason, we ensure a fair and quantitative comparison by selecting the previous methods using the same dataset base on the same assessment method (DSC). Table 5 summarizes different infarct lesion segmentation methods applying the ALATS dataset. The details about the experimental setups of each method and reported performance (DSC) are also described in the table [25]. From this table, we can prove that our proposed infarct lesion segmentation method can provide a slightly higher DSC value compared to the previous methods. As stated in [24], the DSC of the human expert gold standard can be considered in the range of 0.67. Therefore, the DSC of our proposed segmentation method is quite close to that of the gold standard.

## 5. Conclusions

In this study, we have proposed a method for automated segmentation of infarct lesions from T1 weighted brain MRIs. For technical contributions, our study brings three major ideas: (1) applying variational mode decomposition (VMD) for preprocessing of input MRI volumes, (2) dividing the preprocessed MRIs into overlapped patches together with the associated reference numbers, and (3) segmenting the infarct lesions using three-dimensional U-Net. The first contribution, VMD, decomposed the input MRIs into different images by highlighting different spectral bands, which are the key indicators to extract silent image features from infarct lesions. We suppressed the non-stroke (non-infarct) lesions inside the brain by analyzing the spectral characteristics of decomposed images. This contribution helps to reduce the computation effort and time because the segmentation model does not need to work on non-infarct lesions. Moreover, it can implicitly relieve the overfitting and data imbalance problems that specifically occurred due to the high numbers of non-infarct lesions. The second contribution, overlapped patches strategy, is also applied as a preprocessing aiming to reduce the workload of the 3D U-Net based segmentation. Instead of direct inputting the whole MRI volume (197 × 233 × 189 mm^3^), overlapped patches are generated and fed into the U-Net model. Training the U-Net using multiple small patches is also an implicit way of data argumentation and it makes the model more robust. Moreover, thanks to the use of VMD in preprocessing, we can get rid of some empty patches from segmentation. This also considerably reduces the computational effort. Besides, as our proposed overlapped patches strategy records the reference number of the patches, we can easily finetune the final segmented volumes in the postprocessing step. Finally, the last contribution is the development of a three-dimensional U-Net using the extracted patches to segment the infarct lesions. U-Net model performed patch-wise segmentation, and its outputs are postprocessed to get the full-size segmented volumes.

Our proposed method is developed and evaluated using 239 T1 weighted MRI scans (with a total of 430 infarct lesions) from a standard dataset called ATLAS. Based on the experimental results, our method has achieved a mean DSC (0.6684), IoU (0.5022), and ASSD (0.3932), respectively. Moreover, empirical comparison with some previous popular works established using the same dataset also proved that our proposed method can provide preferable segmentation performance. Thus, we believe that our proposed automated infarcted lesion segmentation method can be applied as an adjunct tool to relieve the complications of manual lesion segmentation and assist in providing timely diagnosis decisions and treatments for patients. However, as a major limitation, our proposed work is a unimodal and focus on T1 weighted MRI scans. Hence, its efficacy can be further improved using multimodal MRI scans. Moreover, we have a great interest in improving the performance of our segmentation model using VMD with a combination of the modernized architecture of U-Net. Thus, we believe that the proposed idea of this paper will also be a great help for readers to get future research directions in the automatic diagnosis of any other neurological diseases.

## Figures and Tables

**Table 1:** Data preparation of the proposed method.

Partitions	Number of Scans	Subject ID	Number of Lesions
Training	143	c0003 to c0007 (c0007s0020t01)	268
Validation	48	c0007 (c0007s0021t01) to c0010(c0010s0009t01)	88
Testing	48	c0010(c0010s0009t02) to c0011(c0011s0015t01)	74

**Table 2:** Parameter values for VMD.

Names of the Hyperparameters	Selected Values
Bandwidth constraint (∝)	1000
Number of modes (K)	5
Lagrangian multipliers dual ascent time step (s)	0.5
Tolerance (τ)	K × 10^−6^
Estimated mode center-frequencies (Ω)	1

**Table 3:** Hyperparameter values for proposed 3D U-Net.

Names of the Hyperparameters	Selected Values
Batch size	16
Drop-out rate	0.2
Learning rate	0.001
Number of iterations (Epochs)	20
Optimizer	Adam
Loss function	Dice loss

**Table 4:** Average assessment measurement values.

Assessment Measures	Mean (Std) Values
Intersection over Union (IoU)	0.5022 (±0.0206)
Dice similarity coefficient (DSC)	0.6684 (±0.0187)
Average symmetric surface distance (ASSD)	0.3932 (±0.1475)

**Table 5:** Comparison of performance measurements with state-of-art methods.

Method	X-Net [21]	(CLCI-Net) [22]	2.5D CNN [24]	D-UNet [20]	3D-Res-UNet [25]	Proposed Method
Data Source	ATLAS	ATLAS	KF & MCW (Train) ATLAS (Test)	ATLAS	ATLAS	ATLAS
Number of Samples	229	220	99 (54 from ATLAS)	229	239	239
Data Split Ratio (Train, validation, test)	5-fold cross validation	55, 18, 27	100 for testing	80, 20, 0	76, 11, 13	60, 20, 20
Input size (Height × Width × Depth)	192 × 224 × 1	176 × 233 × 1	192 × 224 × 192	192 × 4 × 192	144 × 172 × 168	197 × 233 × 189
Base Architecture	2D U-Net	2D U-Net	2.5D U-Net	3D U-Net	3D U-Net	3D U-Net
Loss function	Dice loss, cross-entropy	Dice loss	Dice loss	Dice loss, focal loss	Dice loss, cross-entropy	Dice loss
Reported DSC	0.49	0.58	0.54	0.54	0.64	0.6684 *

* The highest DSC value was highlighted in bold.

## Data Availability

Brain MRI scans applied in this study can be accessed via https://doi.org/10.3886/ICPSR36684.v3 or https://www.icpsr.umich.edu/icpsrweb/ADDEP/studies/36684 (accessed on 17 September 2019) with appropriate data usage agreement.
